# Impact of different sowing dates and irrigation levels on NPK absorption, yield and water use efficiency of maize

**DOI:** 10.1038/s41598-023-40032-9

**Published:** 2023-08-10

**Authors:** Ahmed S. D. Abaza, Ayman M. S. Elshamly, Mona S. Alwahibi, Mohamed S. Elshikh, Allah Ditta

**Affiliations:** 1 Water Studies and Research Complex, National Water Research Center, Cairo, Egypt; 2 Department of Botany and Microbiology, College of Science, King Saud University, 11451 Riyadh, Saudi Arabia; 3 Department of Environmental Sciences, Shaheed Benazir Bhutto University Sheringal Dir (U), Dir Upper, KPK Pakistan; 4 School of Biological Sciences, The University of Western Australia, Perth, WA 6009 Australia

**Keywords:** Climate change, Environmental impact

## Abstract

Upper Egypt experiences high temperatures during summer and low temperatures during winter, which significantly impacts the sowing dates of maize in this region. The productivity of maize crops and water use efficiency can be greatly affected by water stress and sowing dates (SDs). Therefore, it is crucial to determine the optimal irrigation level and SDs based on local conditions. To assess the effects, two irrigation levels were employed: (1) control (full irrigation water applied) and (2) 70% of irrigation water. Field experiments were conducted at the National Water Research Center's water studies and research complex station in Toshka. The aim was to evaluate two irrigation levels (full and limited irrigation) across five SDs (early: mid-February and March, normal: mid-June, and late: mid-August and September) in both 2019 and 2020, in order to identify the ideal sowing date (SD) and irrigation level. The normal SD resulted in an increased the growth season length between plant emergence and maturity. Conversely, the late SD reduced the number of days until plant maturity, resulting in higher grain yields and water use efficiency (WUE). Notably, the SD in September, coupled with the 70% irrigation level, yielded the highest productivity and WUE, with a productivity of 7014 kg ha^−1^ and a WUE of 0. 9 kg m^−3^. Based on the findings, it is recommended that regions with similar conditions consider cultivating maize seeds in September, adopting a 70% irrigation level, to achieve optimal N uptake, growth traits (plant height, ear length, ear weight, number of rows per ear, and grain index weight), yield, and WUE.

## Introduction

Water plays a critical role in agricultural production and is one of the most valuable resources, with agriculture being the largest consumer of water^1^. The challenge of climate change affects various sectors of society, including agriculture, water resources, and irrigation water demand^2,3^. Agriculture, as the primary source of sustainable food, is significantly impacted by climate change and extreme weather events, such as temperature fluctuations, irregular precipitation, and water scarcity^4,5^. These changes have adverse effects on productivity, water resources, and the nutritional quality of agricultural products, leading to fluctuations in food production and posing a threat to the constant and sustainable production of cereal-based food^6^. Therefore, Baum Mitch et al.^7^ pointed out that climate change has affected maize optimum planting date and an increase of 1 °C in average temperature increases the growth season period by ten days while the optimum planting date changed by − 2 to + 6 days, according to the cultivar. Hence, adaptation strategies for agricultural systems are essential to address the consequences of climate change on irrigation water demand^8^.

Water stress is an inevitable factor that exists across different environments, disregarding borders and providing no clear warnings. It hampers crop yield, quality, and biomass production^9^. Water stress has detrimental impacts on plants, including delayed growth, reduced photosynthesis, and inhibition of essential biochemical processes^10^. In response to water stress, plants employ various strategies to protect themselves, ranging from essential to auxiliary reactions^11^. These responses enable plants to adapt in the short term to cope with temporary water stress. However, severe or prolonged water stress can adversely affect plant growth and yield^12^. Additionally, the effects of water stress on agriculture are compounded by limited water resources and an increasing global demand for food due to alarming population growth^13^. Consequently, Soares et al.^2^ emphasized the need for sustainable production to meet the demands of a growing global population. Previous researchers study the impact of various irrigation levels on maize yield and WUE^14,15^. They demonstrated that maize productivity was negatively correlated with irrigation levels. Where, Inadequate or excessive irrigation water quantities will limit maize yield and WUE^16,17^. Elshamly^15^ observed that water regime negatively affected the uptake of P, resulting in reduced root efficiency, growth and other vegetative and yield traits, while N, K and protein content increased. Therefore, Kulczycki^18^ concluded that “although maize as another C_4_ plant is highly efficient in WUE, it remains susceptible to the impacts of water availability”.

Liaqat et al.^19^ discovered that the SD significantly influenced crop phenology, specifically tasseling and silking. The timing of planting plays a crucial role in maximizing maize yield and determining grain quality^20^, which has led to extensive research on the response of maize yield to different SDs^21^. Furthermore, the growing environment can also impact the quality and composition of maize kernels^22^. Djaman et al.^23^ observed a notable effect of SD on maize yield and its components. Where early SD enhance the productivity of maize grain and quality as plants reach their physiological maturity before the onset of low fall or winter temperatures^23^. In the kharif and summer seasons, delayed seeding resulted in reduced days to tasseling, silking, length of harvest, ratio of leaf fresh weight to total silage weight, and ultimately grain yield^24^. In this concern, Parker et al.^25^ demonstrated that early SD of maize was correlated with potentially under optimal soil and climatic conditions, while late SD exposes maize plants to a decrease growing season period, low temperatures degrees, and low-income solar radiation. Moreover, suboptimal environmental conditions can limit seed production through asynchronous processes (e.g., adverse impacts on crop growth rate and phenology, hindering the uptake of macronutrient and the synthesis processes^15,26^.

In Egypt, the recommended sowing period for maize falls between May 20 and May 30^19,27^. Similarly, studies by^28–30^ have indicated that maize sown during the second week of August in arid regions such as Toshka district positively enhances maize yield and its components.

Given Egypt's water scarcity and the significance of maize as an oil and fodder crop, this study aims to determine the optimal water requirements and SD for maize cultivation in arid areas.

## Materials and methods

### Experimental site

In the south of Egypt, an open field experiment was conducted at the experimental farm of water studies and research station, Egypt, through the two successive seasons of 2019 and 2020, to study the effect of defining the optimum and planting date and irrigation level under extremely arid conditions. The soil samples were collected from the depth of 0–30 and 30–60 cm. The soil samples were stored in a box and taken to the laboratory, where they were air-dried at room temperature. Thereafter, these soil samples were crushed and sieved through a 2-mm sieve to remove any gravel and coarse plant residues and prepared to determine the physicochemical properties and water status. Using Systronics 372 pH/EC/ TDS/Salinity meter at 25 °C, soil pH and electrical conductivity (EC) were determined and following the protocol elaborated by Janke et al.^31^. Soil particle size distribution was determined using the pipette method, whereas soil textural classes were determined by using the methods developed by USDA Soil Survey Staff^32^. The texture of the experimental soil is sandy soil. While the remaining soil samples were passed through a 0.5 mm sieve and used for determining the rest physical and chemical properties which are given in Table 1, following standardized methods Estefan et al.^33^. In the experimental site, the source of irrigation water is groundwater through a well, according to the analysis quality of the water, it has been classified as C_2_S_1_^34^.

**Table 1:** The physicochemical properties of soil at the experimental site, Egypt during the growing seasons of 2019 and 2020.

Parameter	Unit	Value		Analytical method used
		0–30	30–60	
Mechanical analysis				
Sand	%	92.95	93.88	International pipette method Estefan et al.^33^
Silt	%	3.75	4.06	
Clay	%	3.30	2.06	
Texture	Sand	Soil Survey Staff^32^		
Chemical analysis				
pH (1:5)		7.90	7.8	Systronics 372 electrode pH meter in 1: 5 soil–water suspension Janke et al.^31^
Electrical conductivity (EC) 1:5 at 25 °C	ds m^−1^	0.60	0.39	Systronics 372 electrode EC/ TDS/Salinity meter in 1: 5 soil–water suspension Janke et al.^31^
CaCO_3_	%	8.30	6.0	Titration method Estefan et al.^33^
Available Nitrogen (N)	mg kg^−1^	13.0	20.0	Kjeldahl's method Estefan et al.^33^
Available Phosphorus (P)	mg kg^−1^	6.5	6.0	Spectrophotometer at 410-nm wave length Estefan et al.^33^
Available Potassium (K)	meq l^−1^	0.3	0.2	Flame Photometer Estefan et al.^33^
Magnesium cations (Mg)	meq l^−1^	0.6	1.0	EDTA-disodium salt solution
Sodium cations (Na)	meq l^−1^	1.0	1.1	Flame Photometer Estefan et al.^33^
Calcium cations (Ca)	meq l^−1^	1.6	1.0	Titration method Estefan et al.^33^
Chloride anions (Cl)	meq l^−1^	1.2	1.0	Mohrs’s titration Estefan et al.^33^
Bicarbonate anions (HCO_3_)	meq l^−1^	0.3	0.2	Titration method Estefan et al.^33^
Sulfate anions (SO_4_)	meq l^−1^	1.8	2.0	Barium sulfate (BaSO_4_) precipitation Estefan et al.^33^
Organic matter	%	0.01	0.1	Walkley–Black method Estefan et al.^33^
Water status				
Saturation percent	%	22.0	27.0	Gravimetric method Vaz et al.^42^
Field capacity	%	11.9	13.8	
Wilting point	%	4.9	4.8	

Each value represents the mean of three replications.

### Meteorological data

The studied area lies in the hyper-arid with a mild winter and a hot summer (the mean temperature of the hottest month is 18–34 °C), with the lowest rainfall^35^. Tables 2 and 3 presented the averages of meteorological data, which have been collected from the Toshka weather station during the growing seasons.

**Table 2:** Weather data from the experimental site throughout the period of (January to December) during the 2019 growing seasons.

Month	RH_avg_	SR	Temperature		WS	AP	E pan	P	ST_avg_
			Tmax	Tmin					
January	32.5	141.5	23.7	8.06	2.8	993.7	5.46	0	20.4
February	31.2	163.3	24.8	7.1	3.4	993.0	5.04	0	24.0
March	24.4	216.5	28.8	12.8	3.1	991.6	6.44	0	25.9
April	21.38	241.5	34.4	17.7	3.5	987.6	8.19	0	30.8
May	15.3	287.0	41.3	23.3	3.1	983.9	9.31	0	33.6
June	17.5	312.2	43.2	27.2	3.1	983.0	11.34	0	35.4
July	17.8	310.0	42.1	25.8	2.8	981.8	11.2	0	37.7
August	23.6	231.7	40.8	26.6	2.9	983.0	11.83	0	38.2
September	22.9	221.5	36.8	24.5	3.7	984.0	9.94	0	34.7
October	20.4	227	37.7	23.8	3.4	986.0	5.39	0	30.9
November	33.7	154.4	31.4	16.5	2.7	985.0	5.39	0	26.4
December	40.6	135.5	25.0	10.6	3.0	993.0	4.48	0	23.2

RH_avg_ average relative humidity (%), SR solar radiation (watt m^2^), Max maximum temperature (°C), Min minimum temperature (°C), WS wind speed (m s^−1^), AP atmospheric pressure (millibars), E pan evaporation (mm), P precipitation (mm), and ST_avg_ soil temperature (°C). The meteorological data were obtained from Toshka agrometeorological station, Egypt. Values are the mean of replicates ± standard errors.

**Table 3:** Weather data from the experimental site throughout the period of (January to December) during the 2020 growing seasons.

Month	RH_avg_	SR	Temperature		WS	AP	E pan	P	ST_avg_
			Tmax	Tmin					
January	33.1	161	24.3	8.8	2.9		5.8	0	21.1
February	35.4	181.4	25.1	9.4	3.5	7.0	7.0	0	24.9
March	26.1	213.6	30.9	14.9	2.9	8.3	8.3	0	28.9
April	22	244.6	34.15	18.2	3.2	9.1	9.1	0	31.7
May	19	286	39.6	22.8	3.4	9.3	9.3	0	34.8
June	16.6	327	41.6	24.7	3.4	9.3	9.3	0	36.6
July	19.1	309.5	41	25.4	2.9	10.2	10.2	0	37.7
August	24.8	230.2	41.3	27.1	2.75	12.1	12.1	0	39.2
September	20.1	217	36.7	26.6	3.3	10.5	10.5	0	35.9
October	17.9	232	36.4	23.7	3	6.0	6.0	0	31.7
November	37.1	158.2	32.7	18.3	2.8	6.1	6.1	0	27.9
December	43.4	144	26.2	11	3.2	5.0	5.0	0	24.3

RH_avg_ average relative humidity (%), SR solar radiation (watt m^2^), Max maximum temperature (°C), Min minimum temperature (°C), WS wind speed (m s^−1^), AP atmospheric pressure (millibars), E pan evaporation (mm), P precipitation (mm), and ST_avg_ soil temperature (°C). The meteorological data were obtained from Toshka agrometeorological station, Egypt. Values are the mean of replicates ± standard errors.

### Experimental design and agronomic practices

In order to accomplish the purpose of the current study under drip irrigation system, a split-plot design with five replicates was chosen, whereas the SD early (mid-February—date_1_), normal (March-date_2_ and mid-June—date_3_) and late (mid-August—date_4_ and September—date_5_) in 2019 and 2020) were allocated in the main plot, and two of the irrigation water levels, i.e., 100 and 70% of the water requirements. From the field crops institute, agricultural research center, Egypt, a triple hybrid *Giza 352* of maize seeds were obtained. This cultivar is recommended as a high production commercial cultivar. Moreover, this cultivar and the implemented methods in the current research complied with international, national, and institutional guidelines and legislation. The fertilization management and the field practices were implemented as recommended by the Ministry of Agriculture in Egypt for the newly reclaimed soils. The cultivar of maize was resistant hybrid to late wilt and the harvesting takes place 110–120 days after sowing. At a rate of 35 kg ha^−1^, two maize seeds were sown in hills on one side of the dripper’s jet with a spacing between the maize plants of about 20 cm, while the spacing between rows was 50 cm, with a depth of 5 cm, and the length of lateral lines was 4 m. After 2 weeks of emergence, the plants were thinned to maintain one plant per hill and a population density at 10 plants m^−2^ (100,000 plants ha^−1^). The plot size was 5 × 3.5 m, accordingly, the experimental work involved 50 plots {2 irrigations levels × 5 SDs × 5 replicates}. Additionally, the experimental units were bounded with a buffer zone (2 m width) to prevent interactions. Corn plants were irrigated by drip irrigation system and each irrigation plot had a pressure gauge valve to maintain the operating pressure at 1 bar. A flow meter with a discharge of 25 m^3^ h^–1^ was employed to measure the quantity of targeted amount of irrigation water for each irrigation regime.

### Calculations related to irrigation

#### Crop evapotranspiration (ET_c_)

By entering the weather data that were obtained from the Toshka agrometeorological station, in CROPWAT which is a software package using Fao Penman–Monteith^36^, to calculate ET_o_ on a daily basis from the measured climatic data. Then ET_c_ was calculated according to the following equation:

1 $${\text{ET}}_{{\text{c}}} = \, \left( {{\text{ET}}_{{\text{o}}} \times {\text{ kc stage}}} \right)$$

where ET_c_ = the crop evapotranspiration (mm). ET_o_ = the reference evapotranspiration (mm). k_c_ = the crop coefficient (which was according to^37^ equaled 0.24, 1.04, and 0.58 for Kc_ini_, Kc_mid_, and Kc_end_).

The calculation of the irrigation water requirements (100% Ir) was according to the equation of Abd El-Wahed and Ali^38^ as follows

2 $$\mathrm{Ir }=\frac{\mathrm{A}\times \mathrm{ Etc}\times \mathrm{Ii}\times \mathrm{Kr}}{\mathrm{Ea }\times 1000 \times \left(1-\mathrm{Lf}\right)}$$

where Ir = the irrigation water requirements (mm). A = the plot area (m^2^), Et_c_ = the crop evapotranspiration (mm). Ii = the intervals between irrigation (day), Kr = the coverage coefficient (Kr = (0.10 + Gc) ≤ 1) to Abd El-Mageed et al.^39^, Gc is ground cover. Lf = the leaching factor 10% (since soil electrical conductivity is low, Lf was neglected). Ea = irrigation system efficiency was calculated for the 60 cm soil depth according to Hiekal^40^ as mean values of 3rd, 7th, 17th, and 25th irrigation events according to the equation

3 $$\mathrm{Ea}=\frac{Ws}{Wf}\times 100$$

where Ea = water application efficiency (%). Ws = amount of water stored in the root zone (m^3^ ha^−1^), which was calculated according to Aiad^41^. Wf = amount of water delivered to each plot (m^3^ ha^−1^).

Before the study was started, soil water parameters were measured by gravimetric method as mentioned by Vaz et al.^42^, then the declinations in the soil moisture till it reaches to 50% of available water were recorded, which previous studies demonstrated that was the critical limit on yield. Accordingly, based on this knowledge the irrigation was every 2 days. Furthermore, the applied water irrigation amounts of (70% Ir) treatment were proportionally obtained from the (100% Ir) treatment. The ET_c_ and Ir calculated amounts that are applied to maize crops in the different growth stages during the growing seasons of 2019 and 2020 are demonstrated in Table 4

**Table 4:** The mean crop evapotranspiration and irrigation water applied for maize at different sowing dates during the growing seasons of 2019 and 2020.

	Growth stages				Total
	Seedling (VE-V5)	Vegetative (V6-VT)	Flowering (R1–R5)	Maturation (R6)	
Date_1_ (February)					
Growing season length (days)	24	24	40	19	107
ET_C_ (mm)	76.1	199.3	294.9	69.3	639.6
Irrigation system efficiency%	85	85	85	85	
Ir (mm)	89.5	234.5	346.9	81.5	752.4
Ir_100_ (m^3^ ha^−1^)	895.0	2345.0	3469.0	815.0	7524.0
Ir_70_ (m^3^ ha^−1^)	626.5	1641.5	2428.3	570.5	5266.8
Date_2_ (March)					
Growing season length (days)	25	29	50	11	115
ET_C_ (mm)	102.2	439.7	471.3	93.3	1106.5
Irrigation system efficiency%	86	84	85	85	
Ir (mm)	118.8	523.5	554.5	109.8	1306.6
Ir_100_ (m^3^ ha^−1^)	1188.0	5235.0	5545.0	1097.7	13,065.7
Ir_70_ (m^3^ ha^−1^)	831.6	3664.5	3881.5	768.4	9146.0
Date_3_ (June)					
Growing season length (days)	22	25	53	12	112
ET_C_ (mm)	122.0	476.4	548.6	172.6	1319.6
Irrigation system efficiency%	85	83	84	84	
Ir (mm)	143.5	574.0	661.0	205.5	1584.0
Ir_100_ (m^3^ ha^−1^)	1435.0	5740.0	6610.0	2055.0	15,840.0
Ir_70_ (m^3^ ha^−1^)	1004.5	4018.0	4627.0	1438.5	11,088.0
Date_4_ (August)					
Growing season length (days)	22	22	45	18	107
ET_C_ (mm)	127.8	261.7	358.9	182.2	930.6
Irrigation system efficiency%	84	84	85	85	
Ir (mm)	152.1	311.5	422.2	214.4	1100.2
Ir_100_ (m^3^ ha^−1^)	1521.0	3115.0	4220.0	2144.0	11,000.0
Ir_70_ (m^3^ ha^−1^)	1064.7	2180.5	2954.0	1500.8	7700.0
Date_5_ (September)					
Growing season length (days)	20	21	38	22	101
ET_C_ (mm)	112.6	224.9	343.6	97.9	779.0
Irrigation system efficiency%	85	85	86	86	
Ir (mm)	132.5	264.6	399.5	113.8	910.4
Ir_100_ (m^3^ ha^−1^)	1325.0	2645.7	3995.0	1138.0	9103.7
Ir_70_ (m^3^ ha^−1^)	927.5	1852.0	2796.5	796.6	6372.6

ET_C_ crop evapotranspiration, mm millimeter, m^3^ h^−1^ cubic meter per hectare, VE emergence stage, VT tasseling stage, R1 silking stage, R5 dent stage, Ir_100_ (applying 100% of irrigation water requirements), Ir_70_ (applying 70% of irrigation water requirements).

#### Water use efficiency (WUE)

The WUE was calculated using the following formula:

4 $${\text{WUE}} = { }\left( {\frac{{\text{Y}}}{{{\text{ETc}}}}} \right)$$

where WUE = water use efficiency (kg m^−3^), Y = yield (kg ha^−1^) and ET_c_ = seasonal actual evapotranspiration (m^3^ ha^−1^).

### Measurements

At the harvest, the following measurements were recorded on five samples randomly selected from each plot: Average plant height (cm)—Average ear length (cm)—Average number of ears plant^−1^—Average weight of ear (g)—Average number of row ear^−1^—Average 1000 grain index weight (g) adjusted to 15.5% moisture content—grain yield was determined for each plot then converted to Kg ha^−1^.

### Macronutrient analysis

#### Measurements of nitrogen (N), phosphorus (P), and potassium (K)

At the harvest, three maize kernels of each plot were dried at 65 °C in an air-forced oven for 48 h and then ground into a powder. The samples were digested by a mixture of H_2_SO_4_/H_2_O_2_. Using Micro-equipment, Kjeldahl's as described in^43^. In contrast to the measurement of P using a UV–VIS spectrophotometer and the determination of K with a flame photometer, as outlined by^44,45^.

#### Estimation of protein, total carbohydrates, fiber and oil content

Total carbohydrates were determined as described by El-Katony et al.^46^. The protein percentage was estimated by multiplying the content of N in grains (%) with a coefficient of 6.25^47^. Fiber was determined according to the procedure of^48^. On the other hand, oil content in corn grain was measured by the following formula as described by Bai et al.^49^:

$$\mathrm{Oil\%}=\frac{\mathrm{Final weight}-\mathrm{Initial weight}}{\mathrm{Total samples weight}}\times 100$$

### Statistical analysis

Analysis of variance (ANOVA) was established to determine any statistically significant differences using a package Costat version 6.303. The means were separated through a revised least significant difference (LSD) test at the 0.05 level.

## Results

### Weather conditions during the 2019 and 2020 growing seasons

The daily weather conditions during the 2019–2020 experience period are shown in (Table 2 and 3). Maximum, minimum and mean temperatures increased starting in January 1, 2019 maximum values in June and minimum values at the end of December 2019 at the start of January 2020. A similar trend was seen in 2020. The average monthly air temperature ranged from 16.58 °C (January 2019–2020) to 35.2 °C and 33.14 °C (July 2019–2020). The lowest relative humidity was recorded in May 2019, where it recorded 15.3%, while its lowest value was recorded in 2020 in the same month, the heights relative humidity was recorded in September 2019, where it recorded 40.6%, while its heights value was recorded in 2020 in the same month which was 43.4%. The highest and lowest wind speed in 2019 was recorded in September and November and it was 3.70 and 2.70 m s^−1^, respectively, While the lowest and highest wind speeds were recorded in 2020 in February and August, they were 2.75 and 3.48 m s^−1^, respectively. The average temperature decreased during February, then this average increased during March to reach the highest degree during June, then it began to decrease in August and September 2019. The same occurred in 2020, but the highest average temperatures were recorded in August. The average air temperature from February 2019 to February 2020 was 15.95 °C and 17.27.6 °C, respectively. While the average air temperatures in September 2019 and 2020 were 30.65 and 31.65 °C, respectively. Air temperature for May 2020 was 47.2% higher than May 2019. The date_5_ was also characterized by higher soil temperatures than the rest of the SD.

### Effect of SD on the ETc, Ir and the length of the growing season.

Table 4 represents the effect of SD on the ETc, Ir, and the length of the growing season. The adoption of various SDs in this study affected the ETc, Ir and the length of the growing season. Regarding of various SDs, planting maize seed in the date_2_ SD increased the length of the growing season to (115 days). While the shorter length of the growing season was observed in date_5_ (101 days).

The ETc and Ir values decreased in the early or lately SDs of maize (date_1_, date_4_, and date_5_) then it increased gradually till it reaches the highest values at the normal SDs (date_2_ and date_3_). The maximum ETc and Ir values were observed for date_3_ (1319.6 and 1584.0 mm for ETc and Ir, respectively). And the minimum values were observed for date_1_ (639.6 and 752.4 mm for ETc and Ir, respectively). Overall, it is clear that planting maize seed in the late SDs decreased gradually the ETc, Ir, and the length of the growing season.

### Effect of SD and water levels on the N, P and K

**Figure 1:**
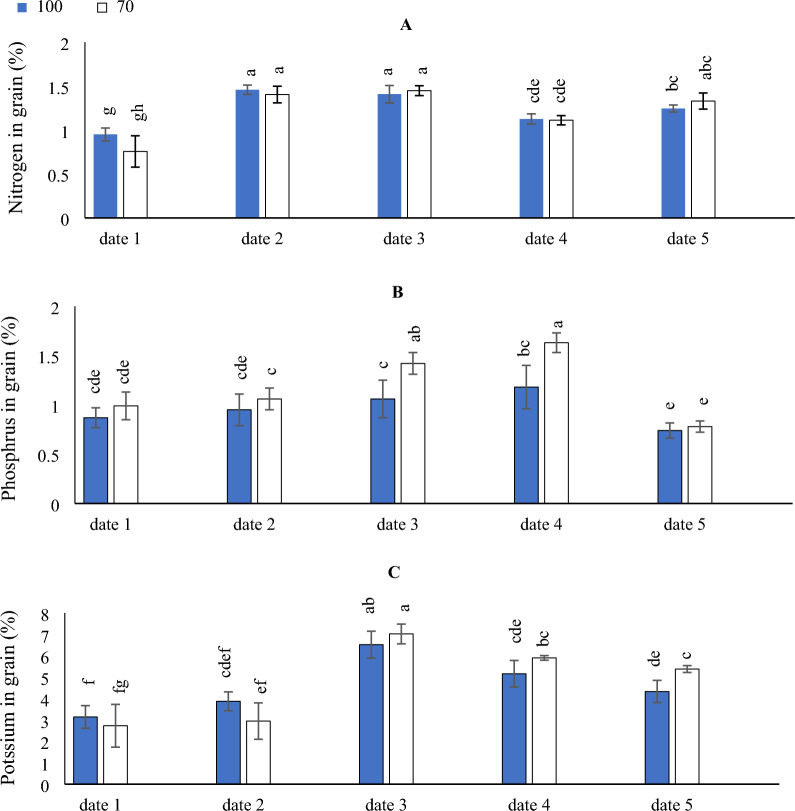
The interactive impact of sowing dates and irrigation levels on nitrogen (**A**), phosphorus, (**B**) and potassium (**C**). Vertical bars represent ± standard error (SE) of the means (n = 5). Bars with different letters are statistically significant at p ≤ 0.05. Abbreviations: date 1 (February); date 2 (March); date 3 (April); date 4 (August) date 5 (September). IR100% (applying 100% of irrigation water requirements—represent full irrigation level); IR 70% (applying 70% of irrigation water requirements—represent limited irrigation level).

As can be observed in (Fig. 1A) the N contents in maize grain under different SD were date_2_ > date_3_ > date_5_ > date_4_ > date_1_. The result showed that when the SD were earlier (date_1_), the concentrations of N in maize grain were decreased. In other hand the contents of N, did not change significantly with drought stress level under various SDs.

On the other hand, by comparing the SD under different irrigation levels, higher N contents were obtained with implementing date_2_ under Ir_100_ & Ir_70_ irrigation levels, although that significantly equaled the implementing of date_3_ under Ir_100_ & Ir_70_ or adoption date_5_ x adopting Ir_70_ irrigation level. Likewise, the lowest N contents were obtained by implementing date_1_ under Ir_100_ & Ir_70_ irrigation levels.

In (Fig. 1B) by comparing the SD under different irrigation levels, higher P contents were obtained with implementing date_3_ and date_4_ under Ir_70_ irrigation levels. Likewise, the lowest P contents were obtained by implementing date_5_ under Ir_100_ & Ir_70_ irrigation levels, although that significantly equaled the implementing of date_1_ under Ir_100_ & Ir_70_ irrigation levels or adoption date_2_ under Ir_100_.

On the other hand, by comparing the SD under different irrigation levels, higher K contents were obtained with implementing date_3_ under Ir_100_ & Ir_70_ irrigation levels. Likewise, the lowest K contents were obtained by implementing date_1_ under Ir_70_ irrigation level (Fig. 1C).

### Effect of SD and water levels on the growth parameters of maize

**Figure 2:**
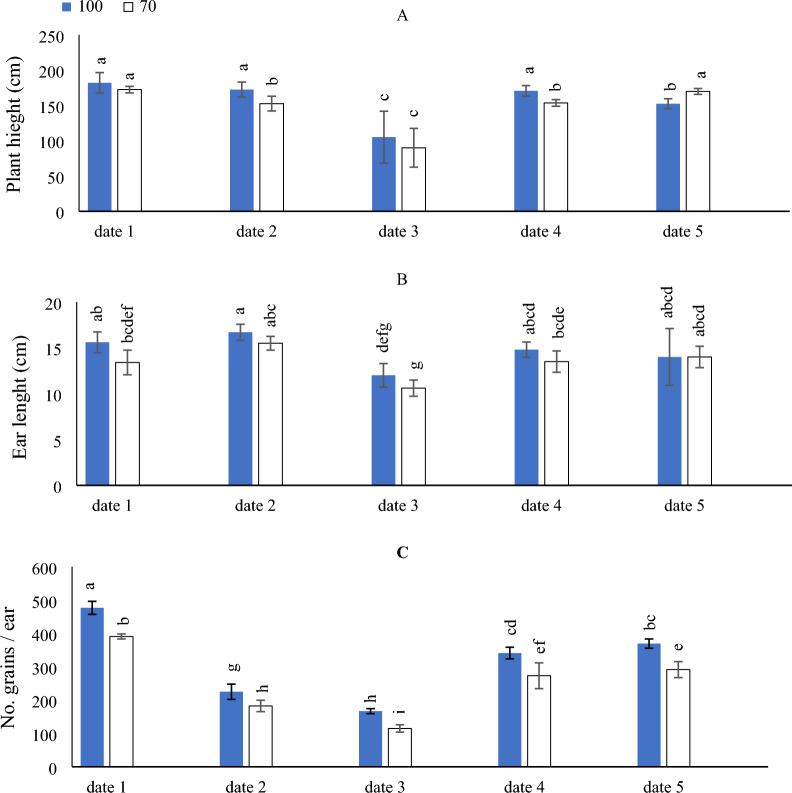
The interactive impact of sowing dates and irrigation levels on maize plant height (**A**), ear length (**B**), number of grains per ear (**C**). Vertical bars represent ± standard error (SE) of the means (n = 5). Bars with different letters are statistically significant at p ≤ 0.05. Abbreviations: date 1 (February); date 2 (March); date 3 (April); date 4 (August) date 5 (September). IR100% (applying 100% of irrigation water requirements—represent full irrigation level); IR 70% (applying 70% of irrigation water requirements—represent limited irrigation level).

In (Fig. 2A) by comparing the SD under different irrigation levels, a higher plant high was obtained with implementing date_1_ under Ir_100_ & Ir_70_ irrigation levels, date_2_ and date_4_ under Ir_100_ irrigation levels, respectively, although that significantly equaled the implementing of date_5_ under Ir_70_. Likewise, the lowest plant high was obtained by implementing date_3_ under Ir_100_ & Ir_70_ irrigation levels.

As illustrated in (Fig. 2B), the results showed that by comparing the SD under different irrigation levels, the adopting of Ir_100_ & Ir_70_ irrigation levels × (date_2_ and date_5_) significantly equaled the adoption of Ir_100_ irrigation level x (date_1_ and date_4_), for attaining the highest ear length in the maize. While the data indicated that the lowest ear length was recorded under the adopting of Ir_100_ & Ir_70_ irrigation levels × date_3_.

By comparing the SD under different irrigation levels, a higher grain number was obtained with implementing date_1_ under the Ir_100_ irrigation level. Likewise, the lowest grains number were obtained by implementing date_3_ under the Ir_70_ irrigation level (Fig. 2C).

**Figure 3:**
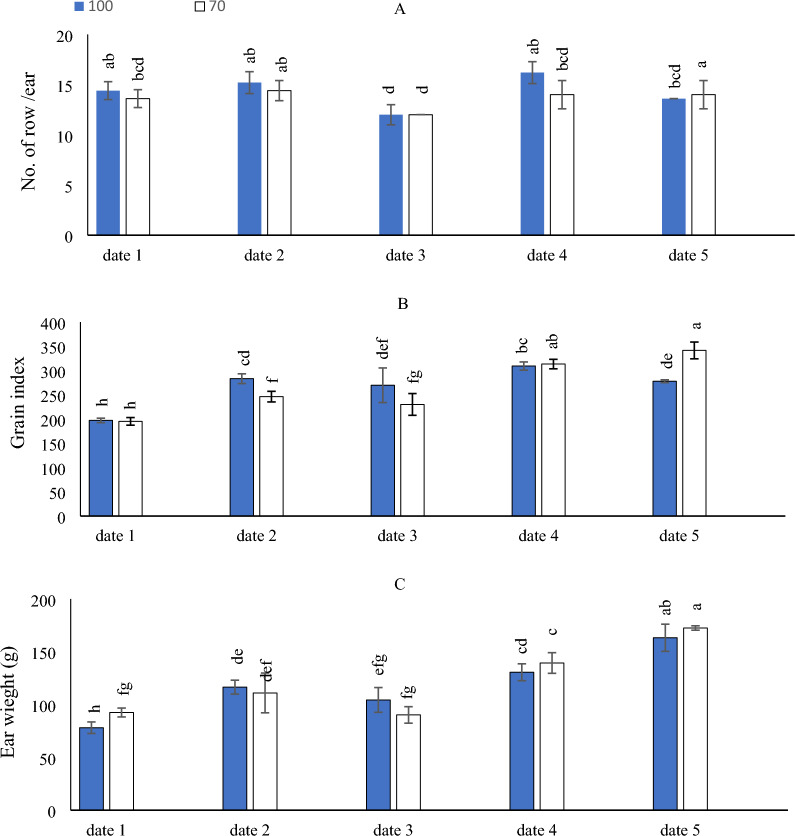
The interactive impact of sowing dates and irrigation levels on number of rows per ear (**A**), grain index (**B**), and ear weight (**C**). Vertical bars represent ± standard error (SE) of the means (n = 5). Bars with different letters are statistically significant at p ≤ 0.05. Abbreviations: date 1 (February); date 2 (March); date 3 (April); date 4 (August) date 5 (September) IR100% (applying 100% of irrigation water requirements- represent full irrigation level); IR 70% (applying 70% of irrigation water requirements—represent limited irrigation level).

As can be seen in (Fig. 3A), to get the best number of rows per maize ear, it is just as effective to adopt the Ir_100_ & Ir_70_ irrigation levels × implementing date_2_, which significantly equaled Ir_100_ irrigation level × the implementation of date_1_ and date_4_ or Ir_70_ irrigation level × date_5_.

The obtained results in (Fig. 3B), indicated that the maximum grain index was achieved through adopting Ir_70_ irrigation level × (date_4_ and date_5_). Likewise, the lowest grain index was obtained by implementing date_1_under Ir_100_ & Ir_70_ irrigation levels.

On the other side, the obtained results indicated that by comparing the examined Ir as seen in (Fig. 3C), it was found that Ir_100_ & Ir_70_ irrigation levels × date_5_ significantly attained the highest ear weight, while the minimum increase of ear weight was observed by the adoption of Ir_100_ irrigation level × date_1_.

### Effect of SD and water levels on protein, total carbohydrates, fiber and oil content of maize

**Figure 4:**
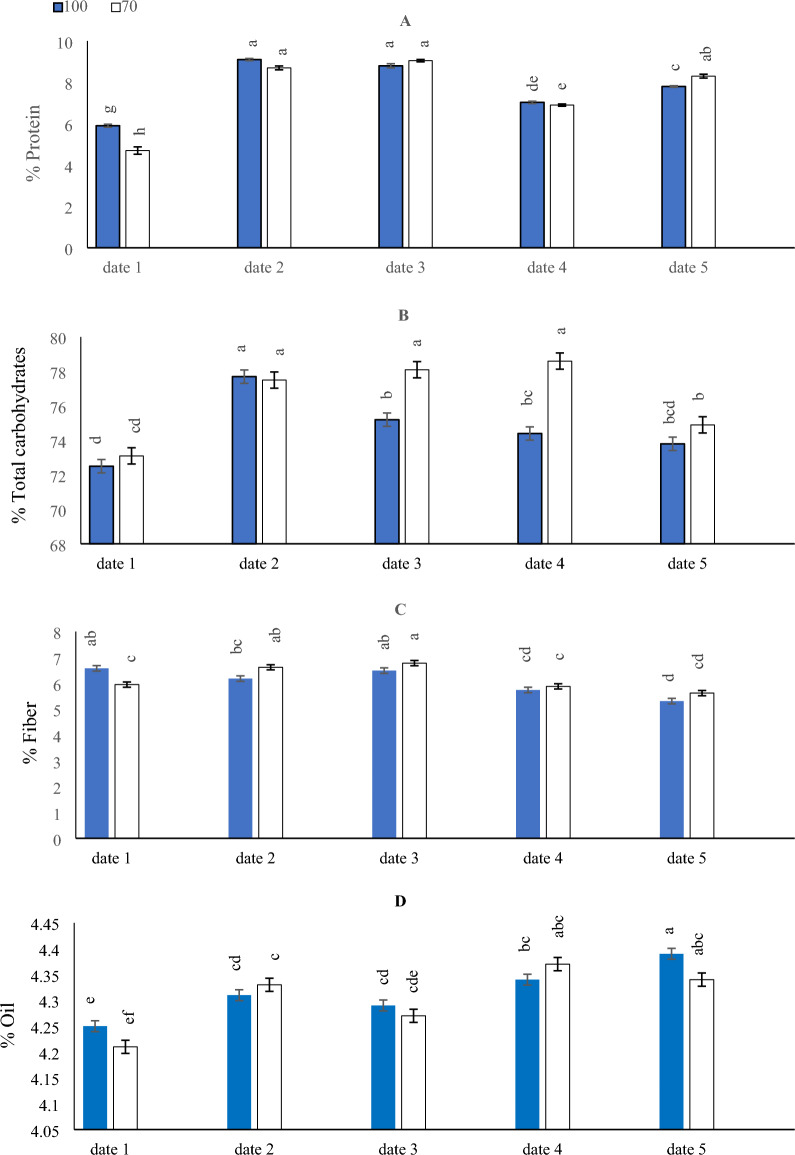
The interactive impact of sowing dates and irrigation levels on protein (**A**), total carbohydrates (**B**), fiber (**C**), and oil contents (**D**) in maize seeds. Vertical bars represent ± standard error (SE) of the means (n = 5). Bars with different letters are statistically significant at p ≤ 0.05. Abbreviations: date 1 (February); date 2 (March); date 3 (April); date 4 (August) date 5 (September) IR100% (applying 100% of irrigation water requirements- represent full irrigation level); IR 70% (applying 70% of irrigation water requirements—represent limited irrigation level).

The effects of SD and water levels on protein, total carbohydrates, fiber and oil content are presented in (Fig. 4). In general, there was a significant difference in the protein content in the date_1_ and date_5_ under Ir_100_ & Ir_70_, but not in the date_2_, date_3_ and date_4_ under Ir_100_ & Ir_70_ treatment (Fig. 4A). The results showed that the adoption of SD date_5_ led to a decrease in protein content under Ir_70_ treatment. However, the adoption of SD date_2_ and date_3_ and the application of Ir_100_ & Ir_70_ treatment resulted in the highest increase in protein content, although this increase was only significantly equal to the adoption of date_5_ + Ir_70_.

As shown in (Fig. 4B), adopting date_2_ + Ir_100_ & Ir_70_ or date_3_ under the Ir_70_ level significantly matched adopting an Ir_70_ water level and implementing SD date_5_ to achieve the highest total carbohydrates in maize seeds. Likewise, the lowest total carbohydrates contents were recorded under date_1_ and the application of Ir_100_ & Ir_70_, which significantly equaled the adoption of date_5_ × Ir_100_ level.

To reduce fiber contents in maize seeds, either planting maize seeds in the late SD (date_5_) under Ir_100_ & Ir_70_ or planting maize seeds in the date_4_ was effective under the Ir_100_ level (Fig. 4C). The highest fiber contents were observed when adopting date_3_ and the Ir_100_ & Ir_70_ levels, although this increase was only significantly equal to the adoption of date_1_ × Ir_100_ or date_2_ under the Ir_70_.

On the other hand, the results indicated that date_1_ under Ir_70_ treatment resulted in significantly lower values of oil content, as seen in (Fig. 4D). The maximum increase in oil content was observed when date_5_ was adopted with the implementation of Ir_100_ & Ir_70_, although this increase was significantly equal to the adoption of date_4_ + Ir_70_.

### Effect of SD and water levels on grain yield and WUE

**Figure 5:**
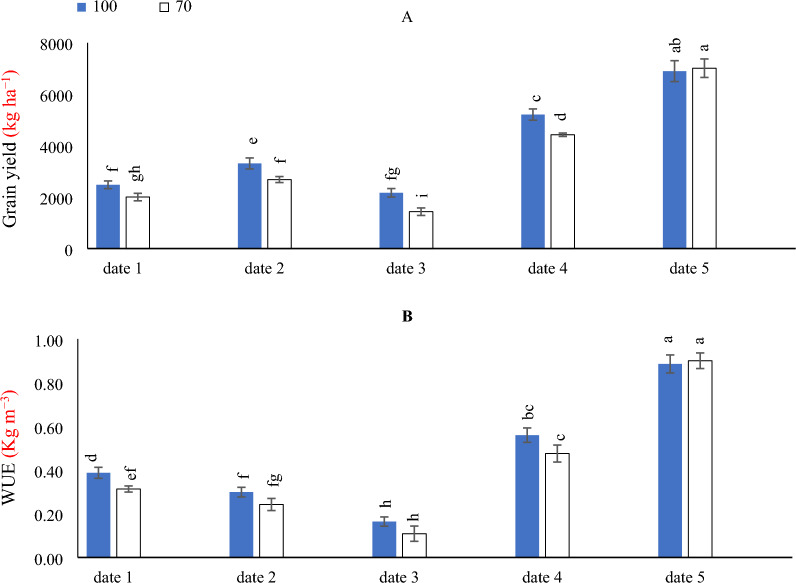
The interactive impact of sowing dates and irrigation levels on maize grain yield (**A**) and water use efficiency (**B**). Vertical bars represent ± standard error (SE) of the means (n = 5). Bars with different letters are statistically significant at p ≤ 0.05. Abbreviations: date 1 (February); date 2 (March); date 3 (April); date 4 (August) date 5 (September) IR 100% (applying 100% of gross irrigation water requirements- represent full irrigation level); IR 70% (applying 70% of gross irrigation water requirements—represent limited irrigation level).

The obtained data that illustrated in (Fig. 5A) demonstrated that by comparing the SD under different irrigation levels, a higher grain yield was obtained with implementing date_5_ under Ir_100_ & Ir_70_ irrigation levels. Likewise, the lowest grain yield was obtained by implementing date_3_ under Ir_70_ irrigation level.

As can be observed in (Fig. 5B), to get the best WUE of maize, it is just as effective to adopt a Ir_100_ & Ir_70_ irrigation levels x date_5_. Nevertheless, the implementation of Ir_100_ & Ir_70_ irrigation levels × date_3,_ causing the lowest decreases in maize WUE.

### Ethics approval and consent to participate

This manuscript is an original paper and has not been published in other journals. The authors agreed to keep the copyright rule.

## Discussion

The SD is a crucial factor that limits maize crop yield and plays a significant role in determining overall yield. Therefore, determining the optimal SD is critical for agricultural production, especially considering the impact of increasing temperatures on crop yield due to shorter growing seasons^50^.

The results obtained from this study revealed that sowing maize seeds on date_2_ and date_3_, with the application of Ir_100_ and Ir_70_, resulted in the highest accumulation of N content. Furthermore, sowing maize seeds on date_5_ under Ir_70_ yielded a significantly similar N content compared to the previous treatment. The lowest accumulated N value was observed when maize seeds were sown on date_1_ under both Ir_100_ and Ir_70_. This could be attributed to the severe stress, including water stress and high temperatures, experienced by maize plants, particularly during the filling stage. In the case of SDs (date_2_ and date_3_), the plants reached the filling stage during the hottest months, such as June and August. As a result, the plants were exposed to high temperatures and water stress, which prompted them to increase N absorption and accumulation to facilitate the production of high molecular components. This can be seen as a protective mechanism against stress and allowed the plants to complete their life cycle quickly. These findings align with previous studies^18,51^. Similarly, Dupont et al.^52^ demonstrated that grain protein (N) percentage was lower under moderate temperatures compared to grains produced under higher temperatures, which is consistent with our findings. However, this response seems to have a critical limitation as crossing a certain threshold can lead to negative impacts. In this context, Klimenko et al.^53^ showed that the absorption and translocation of N in wheat grains decreased under higher temperatures due to a decline in nitrate reductase activity in plants. Under such conditions, plants tend to increase the uptake of other nutrients such as P, K, sulfur, and sodium, which help maintain and protect cell membranes, enhance the antioxidant defense system, and improve osmotic potential, ultimately improving the photosynthetic rate^54^. Nevertheless, the reduction in root growth under higher temperature conditions generally has an adverse influence on the uptake, assimilation, and translocation processes of most nutrients^55^. Therefore, when maize seeds are planted on date_1_, reaching the filling stage around April and early May, the conditions allow maize plants to prioritize vegetative growth over productivity growth. This delay in accelerating the uptake and translocation processes of N occurs after the completion of vegetative growth, as plants face unfavorable temperature conditions, which ultimately results in the lowest accumulation of N in maize grains.

In addition to the accumulation of N content, the accumulation of P in maize seeds appears to be more correlated with the SD rather than the irrigation water regimes used. We observed that the highest P accumulations were achieved with Ir_70_ and the cultivation of maize seeds on date_3_ and date_4_. Conversely, the lowest values of accumulated P were observed on date_5_, although they were comparable to the values obtained from maize seeds planted on date_1_ and date_2_ under Ir_100_. In this context, we hypothesized that under the SDs of date_3_ and date_4_, maize plants experienced a higher air and soil temperature conditions during the most growth stages, which contributed to unfavorable soil moisture content. Under water stress conditions, maize plants undergo different mechanisms to deal with the reduction in applied irrigation. One of these mechanisms is the activation of an effective root system, which involves the penetration of the root system within the soil profile, the modification of root architecture as well as the accumulation of root exudate production, which is consistent with previous studies^56–58^. However, with reduced water applications (Ir_70_), the soil pH increased subsequently affecting negatively on the availability of P^59^. The plants employ increasing root exudates to decrease soil pH and allow root systems increase the absorption of P. Increased accumulation of P in grains improves the synthesis of carbohydrates. Leads to a decrease in water potential, thereby enhancing water uptake by the roots and improving the water status of the plant. Moreover, under water stress conditions (Ir_70_), it can be assumed that plant vegetative aerial parts are less active, leading to reductions in various physiological processes such as transpiration. Consequently, the root system becomes the primary driver of activities, prioritizing nutrient uptake and storage. These mechanisms, coupled with other protective mechanisms adopted by maize plants, contribute to their tolerance and overall performance under water stress conditions. Additionally, it appears that soil temperature was highest on date_3_ and date_4_, resulting in increased P uptake from the rhizosphere under higher temperature conditions compared to date_1_ and date_5_ (low temperature). This finding aligns with previous studies^60^.

On the other hand, the results showed that the accumulation of K in maize seeds reached its highest values when date_3_ was combined with Ir_100_ and Ir_70_, while lowest accumulated K value was observed when maize seeds were sown on date_1_ and date_2_. We hypothesize that severe temperature and evaporation conditions affect plant growth starting from date_3_. As a protective strategy, plants increase the uptake and accumulation of K to cope with these conditions. K has several features that can improve plant water status under water and temperature stress conditions. This is evident in the increased accumulation of K observed on date_3_ under Ir_100_ and Ir_70_. Similar observations have been reported in previous studies^61–63^, although the relationship between K uptake and temperature has a threshold point. For instance^64^, mentioned that the threshold point was at 25 °C, and with further increases in temperature, K uptake decreased. Furthermore^65^, reported that plants have the ability to modify nutrient absorption and accumulation based on the temperature conditions to which their aerial parts or roots are exposed.

Based on the findings of the current study, it is crucial to determine the intended purpose for maize seeds. If the goal is to address malnutrition, cultivating maize seeds on date_3_ under Ir_70_ results in improved seed quality with the highest values of N, P, K, protein, and total carbohydrates. On the other hand, if the aim is to achieve maximum yield, the results demonstrate that planting maize seeds on date_5_ under Ir_70_ leads to the highest maize yield and WUE. From the results, we conclude that on date_5_ when maize adopting the limited irrigation level, the plants are exposed to some degree of water stress that has contributed to a series of successive impacts. Physiologically, this leads to a decrease in soil moisture around the roots leading to an increase in the root penetration and absorption activity. Consequently, water, macronutrient (N), and photosynthesis improved, resulting in enhanced growth traits (plant height, ear length, ear weight, number of rows per ear, and grain index weight), protein, oil content and yield under these conditions (Cai and Ahmed^66^. Additionally, this approach proves beneficial in mitigating the impacts of water stress and conserving significant amounts of irrigation water and increasing WUE in arid climatic conditions. These findings align with previous studies^14,59,67^, that have reported similar enhancements in yield characteristics.

Furthermore, we hypothesize that exposing maize plants to water stress on date_5_ involves interconnected factors that partially mitigate the effects of these conditions. One of these factors is the relatively short duration of water stress that maize plants experience. By sowing maize seeds on date_5_ under Ir_70_, despite the reduction in applied irrigation water, severe adverse impacts are not observed. In this regard^68^, has indicated that plants have the ability to adjust their vegetative and reproductive phenology in response to water reduction, depending on the most favorable period. Moreover, planting maize seeds on date_5_ under Ir_70_ motivates plants to enhance the production process due to the shorter growth period, typically around 100–102 days (average 101 days), as shown in Table 4. Additionally, the presence of combined stress factors, such as temperature and drought, accelerates the absorption and accumulation of N, leading to increased photosynthesis, protein production, and plant growth.

## Conclusion

The findings of this study clearly demonstrate the significant influence of current climatic changes on maize production, nutrient uptake, and yield in arid conditions. However, the specific impacts varied depending on the pattern of water stress implemented. Determining the optimal sowing dates may not always align with practical implementation, as it depends on factors such as available non-cultivated land and the return benefits for farmers.

Nevertheless, if the goal is to increase the concentrations of nitrogen, phosphorus, potassium, protein, and total carbohydrates in maize seeds, earlier sowing dates can be advantageous to benefit from higher seed quality and to create an appropriate window for growing a winter crop. On the other hand, the results highlight the importance of subjecting maize to a certain level of water stress. However, further research is needed to observe the impacts of this practice in different regions.

Considering the current climatic changes, sowing maize seeds in September under 70% of irrigation water requirements appears to be the most favorable approach for achieving optimal N uptake, growth traits (plant height, ear length, ear weight, number of rows per ear, and grain index weight), grain yields, and irrigation water use efficiency in irrigated arid conditions. Additionally, these practices prove beneficial in mitigating the impacts of water stress and conserving significant amounts of irrigation water in arid areas.

## Data Availability

The presented datasets during the current study available from the corresponding author on reasonable request.
